# The Effects of *Helicobacter pylori* on the Treatment Outcomes of Peptic Ulcer in Patients with Liver Cirrhosis: A Systematic Review and Network Meta-Analysis

**DOI:** 10.3390/jcm15062283

**Published:** 2026-03-17

**Authors:** Linhao Zhang, Jinli Lin, Xueying Li

**Affiliations:** 1 Department of Gastroenterology and Hepatology, West China Hospital, Sichuan University, Chengdu 610041, China; 2 Chengdu Women’s and Children’s Central Hospital, School of Medicine, University of Electronic Science and Technology of China, Chengdu 610041, China; lin_jinli64@163.com; 3 West China Second University Hospital, Sichuan University, Chengdu 610041, China; lixueying19920309@163.com

**Keywords:** *Helicobacter pylori*, peptic ulcer, liver cirrhosis, network meta-analysis, eradication therapy

## Abstract

**Background/Objectives:** *Helicobacter pylori* (*H. pylori*) positivity is associated with peptic ulcers in the general population. However, its role in peptic ulcers in cirrhotic patients remains controversial. The impact of *H. pylori* infection status on the treatment outcomes of peptic ulcers in liver cirrhosis was investigated. **Methods**: A systematic review and network meta-analysis was performed. The following databases were searched: Ovid MEDLINE, PubMed, Web of Science, CQVIP, Wanfang, China National Knowledge Infrastructure, chictr.org.cn, and ClinicalTrials.gov. Relevant studies were published up to 31 December 2025. Studies comparing the treatment outcomes of peptic ulcers in liver cirrhosis were included. Patients were divided into three groups: Hp_pos group (including those who failed to eradicate *H. pylori* and who denied eradication therapy), Hp_neg group (*H. pylori*-uninfected individuals), and Hp_Erad group (*H. pylori* tested positive at baseline but was successfully eradicated afterwards). Prospero registration number: CRD42024551260. **Results:** Four prospective studies were eligible. No significant difference was found in the unhealed peptic ulcers or recurrent peptic ulcers, although the Hp_Erad group had the highest values of the surface under the cumulative ranking curve (SUCRA) [(0.773, mean rank: 1.5) and (0.809, mean rank: 1.4), respectively]. **Conclusions:** Based on the available low-quality evidence, this network meta-analysis did not detect a statistically significant benefit of *H. pylori* eradication for ulcer healing or recurrence prevention in cirrhotic patients. These findings should be interpreted as highlighting an evidence gap rather than providing definitive evidence. Further randomized controlled trials are necessary to confirm these findings. Clinical decisions regarding *H. pylori* eradication in cirrhotic patients should be made carefully, weighing the potential benefits against the risks.

## 1. Introduction

*Helicobacter pylori* (*H. pylori*) was discovered over 40 years ago, and currently, the test-and-treat strategy is widely endorsed [1,2]. Although the global prevalence of *H. pylori* is declining, the burden of disease associated with *H. pylori* infection remains substantial [3,4,5]. *H. pylori* positivity is significantly correlated with gastrointestinal diseases, particularly peptic ulcers [6,7]. The lifetime prevalence of peptic ulcers among individuals infected with H. pylori is estimated to be 10% [8]. Peptic ulcers can be fatal if they lead to acute bleeding with a mortality rate of up to 10% [9]. *H. pylori* eradication is essential in the treatment of peptic ulcers, as it prevents relapse and reduces complications of peptic ulcers in the general population [10].

However, for patients with comorbidities such as liver cirrhosis, it is controversial whether *H. pylori* is still the primary contributor to peptic ulcers [11], even though the prevalence of peptic ulcers in cirrhotic patients is higher [12]. In recent consensus and guidelines, no specific recommendation regarding *H. pylori* eradication has been provided for those complicated with liver cirrhosis [10,13,14]. Additionally, some drugs (e.g., clarithromycin) used in typical *H. pylori* eradication regimens may induce liver injury [15], which can be dangerous for cirrhotic patients. The safety and efficacy of *H. pylori* eradication regimens have not been thoroughly investigated in patients with abnormal liver function [16]. Moreover, whether or not to treat *H. pylori* is also a question in the context of increasing antimicrobial resistance in *H. pylori* [17]. Thus, the necessity of *H. pylori* eradication should be investigated in patients with cirrhosis and peptic ulcers.

In this study, we conducted a systematic review and network meta-analysis to investigate the effect of *H. pylori* infection on the treatment outcomes of peptic ulcers in patients with liver cirrhosis. The application of network meta-analysis was necessitated by the classification of patients with cirrhosis into three distinct groups based on their *H. pylori* infection status, which required a comprehensive comparative analysis.

## 2. Materials and Methods

This study was registered with the International Prospective Register of Systematic Reviews (PROSPERO, number: CRD42024551260) and was performed according to the Preferred Reporting Items for Systematic Reviews and Meta-Analyses (PRISMA) guidelines for network meta-analysis [18]. Ethical approval and patient consent were omitted due to the nature of the meta-analysis. The related checklist could be found in Supplemental Material S1: PRISMA NMA checklist.

### 2.1. Eligibility Criteria

Randomized controlled trials (RCTs) and cohort studies published in English and Chinese, evaluating the effect of *H. pylori* on the treatment outcomes of peptic ulcer among patients with liver cirrhosis, were incorporated, without restriction of publication status. The inclusion criteria were as follows: cirrhotic patients were diagnosed with peptic ulcer detected by endoscopy, diagnostic tests for *H. pylori* were performed for these patients, testing for eradication success was repeated for those who underwent eradication therapy, and endoscopy was performed to confirm the treatment outcomes of peptic ulcer for the patients. A peptic ulcer was defined as a mucosal erosive lesion with a crater > 5 mm in diameter, a fibrin-covered base, and a distinct border on the stomach or duodenum. A recurrent peptic ulcer was defined as any repeat endoscopy that identified de novo peptic ulcer after the previous endoscopy confirmed that the original peptic ulcer had healed. The eligible studies were supposed to report the number of patients with unhealed peptic ulcer (primary outcome). Follow-up endoscopy aimed to assess whether ulcer healing should be performed at 4–12 weeks after initial treatment. The patients were divided into three groups according to *H. pylori* infection status and treatment. Hp_pos group: *H. pylori* tested positive throughout the study (including those who failed to eradicate *H. pylori* and who denied eradication therapy). In the Hp_pos group, most patients failed to eradicate *H. pylori*, and proton pump inhibitors were used to further treat peptic ulcers. The rest of the patients in the Hp_pos group denied eradication therapy, and thus only proton pump inhibitors were used to treat peptic ulcers. Hp_neg group: *H. pylori* tested negative at baseline (*H. pylori*-uninfected individuals). Typically, patients in the Hp_neg group were prescribed proton pump inhibitors only. Hp_Erad group: *H. pylori* tested positive at baseline but was successfully eradicated afterwards. Patients from the Hp_Erad group were initially prescribed drugs to eradicate HP, and then proton pump inhibitors were used to treat peptic ulcer further. Articles should be excluded if the full text is not available, or if the treatment outcomes of peptic ulcer were not reported clearly along with the number of patients with persistent *H. pylori* infection after eradication therapy.

### 2.2. Literature Search

Studies involving *H. pylori*, peptic ulcer and liver cirrhosis were searched with several databases, including Ovid MEDLINE, PubMed, Web of Science, CQVIP, Wanfang, China National Knowledge Infrastructure (CNKI), chictr.org.cn and ClinicalTrials.gov. The following terms and their combinations, where appropriate, were used in the search: *Helicobacter pylori*, *Helicobacter pylori* eradication, ulcer, cirrhosis, and hepatogenic ulcer. The references of the articles were also searched. The specific search strategy is provided in the Supplemental Material S2: Search strategy. The last search was conducted on 31 December 2025, without limitations on the publication time. Contact with the study authors to identify additional data was conducted when necessary.

### 2.3. Study Selection

Two reviewers (L.Z. and J.L.) independently evaluated the eligibility of the studies, extracted the data, and assessed the quality of the included studies. Disagreements were resolved by consensus or discussion with a third reviewer (X.L.).

### 2.4. Data Extraction

Two reviewers (L.Z. and X.L.) independently extracted the data. The first author, year of publication, study type, and sample size of each study were documented. The diagnostic methods, regimens of eradication and other treatments, re-evaluation of *H. pylori* after eradication, duration of follow-up, and outcome of ulcer healing were recorded. Other results, such as peptic ulcer recurrence and rebleeding, were also recorded. Disagreements were resolved by consensus or discussion with a third reviewer (J.L.).

### 2.5. Quality Assessment

Each included study was independently assessed by two reviewers (L.Z. and J.L.). Cochrane collaboration tools were used to evaluate randomized controlled trials, and the Newcastle–Ottawa Scale (NOS) was used to assess the quality of nonrandomized controlled studies. According to the Agency for Healthcare Research and Quality standards, an NOS score of 7–9 is indicative of good quality, 4–6 is fair, and 1–3 is poor. The online tool Confidence in Network Meta-Analysis (CINeMA, https://cinema.ispm.unibe.ch/, accessed on 28 July 2024), which is based on the Grading of Recommendations Assessment, Development and Evaluation (GRADE) framework, was used to evaluate the credibility of the results from this network meta-analysis [19].

### 2.6. Geometry of the Network

The geometry of the network included the Hp_pos, Hp_neg, and Hp_Erad groups, with each circle representing a group. The node size was proportional to the pooled sample size. The size of the edge was related to the number of studies included. The connectivity of the network geometry was qualitatively described.

### 2.7. Statistical Analysis

Network meta-analysis was conducted using a frequentist approach with STATA (version 17; STATACorp LLC, College Station, TX, USA). The main analysis was not performed using a Bayesian approach because, owing to the nature of the included studies, inconsistency did not occur [20], and nodesplitting models cannot be generated with R package gemtc, version 4.5.1. Therefore, the consistency model was used. Heterogeneity was quantified using the I-squared statistic. Odds ratios (ORs) were calculated to analyze dichotomous variables with 95% confidence intervals (CIs). Conventional pairwise meta-analysis using a random-effects model was performed to directly compare the effects. Ranking probabilities were assessed using the surface under the cumulative ranking curve (SUCRA), mean ranks, and rankograms. Publication bias was evaluated using Egger’s test and funnel plots. A subgroup analysis was not possible because only a few studies were included.

## 3. Results

**Figure 1:**
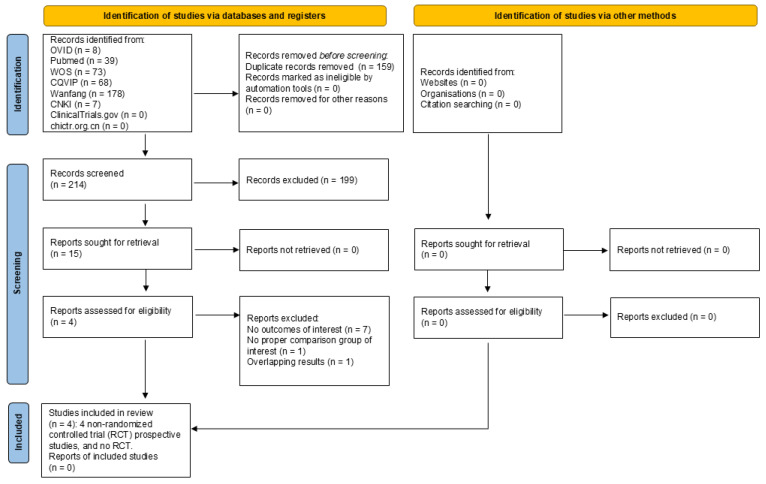
Flow diagram.

The study selection process is illustrated in Figure 1. In total, 214 studies were screened and assessed for eligibility. Four studies were considered eligible for this network meta-analysis [21,22,23,24]. A study by Villalan R was excluded because although the treatment outcomes of peptic ulcer were reported, the number of patients who tested positive for *H. pylori* after eradication therapy was not clearly reported [25]. We attempted to contact the study authors for their data, but no response was received.

### 3.1. Characteristics of the Included Studies

The characteristics and results of the included studies are described in Table 1 and Table 2. All 4 included studies were non-RCT prospective studies, and no RCT was included. The studies conducted by Tzathas C [24], Mitrică D [22], and Lo GH [21] were three-armed studies comparing the Hp_pos, Hp_neg, and Hp_Erad groups, while the study from Mo M [23] was a two-armed study that compared the Hp_pos and Hp_Erad groups. *H. pylori* infection was determined using rapid urease tests and/or histological examinations in all studies. Except for unhealed peptic ulcers after treatment, only recurrence of peptic ulcers was reported in all studies. Other results, such as rebleeding of peptic ulcer, were reported in fewer than three studies and thus could not be analyzed further. In total, 249 patients were analyzed for unhealed peptic ulcers and 244 patients were analyzed for peptic ulcer recurrence.

### 3.2. Quality Assessment and Risk of Bias

Assessment of risk of bias for included studies using the Newcastle-Ottawa Scale (NOS) is presented in Supplementary Table S1, and all four studies were deemed good. CINeMA showed low confidence for both unhealed peptic ulcer and recurrent peptic ulcer due to major concerns regarding imprecision (Supplementary Tables S2 and S3).

### 3.3. Network Diagram

**Figure 2:**
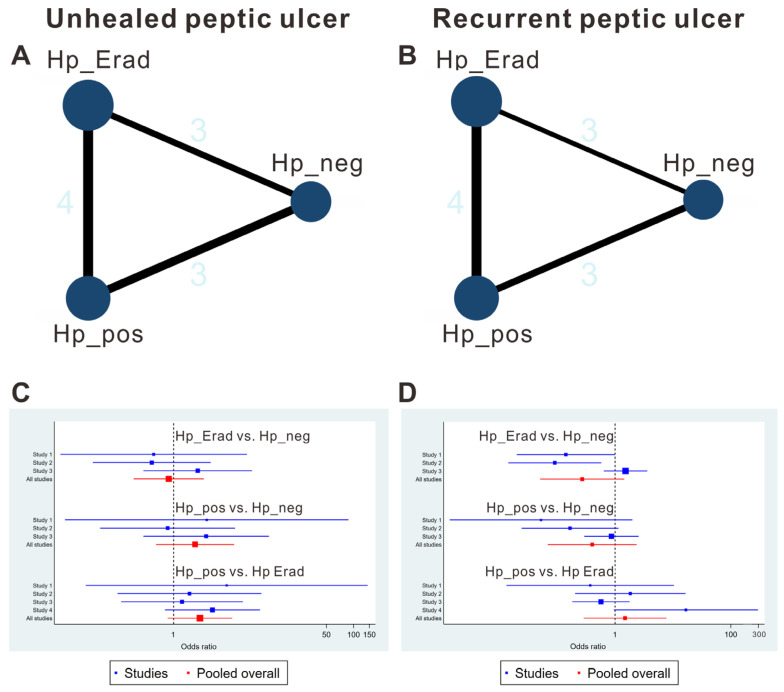
Summary of the results of unhealed and recurrent peptic ulcer. (**A**,**B**) Network plots. (**C**,**D**) Forrest plots.

Regarding unhealed peptic ulcer, 3 studies [21,22,24] reported three-armed comparison (Hp_pos group vs. Hp_neg group vs. Hp_Erad group), and 1 study [23] reported the comparison between Hp_pos group and Hp_Erad group, forming a triangle (Figure 2A). Similarly, the same network geometry was formed for recurrent peptic ulcers (Figure 2B). Networks for these outcomes comprised direct comparisons among the Hp_neg, Hp_pos, and Hp_Erad groups. The contribution of each group is presented in Supplementary Figure S1.

### 3.4. Evaluation of Transitivity

Considering the similarity of the study populations and interventions, we deemed that transitivity was not violated in the included studies.

### 3.5. Assessment of Inconsistency

Inconsistencies could not be statistically tested in this network meta-analysis for both unhealed peptic ulcers and recurrence of peptic ulcers because inconsistencies did not occur [20].

### 3.6. Synthesis of Results

#### 3.6.1. Unhealed Peptic Ulcer

**Figure 3:**
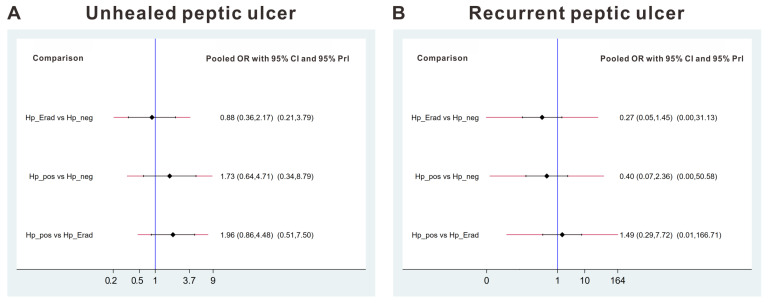
Interval plots for unhealed peptic ulcer (**A**), and recurrent peptic ulcer (**B**). OR—odds ratio. CI—confidence interval. Predictive Interval—PrI.

**Figure 4:**
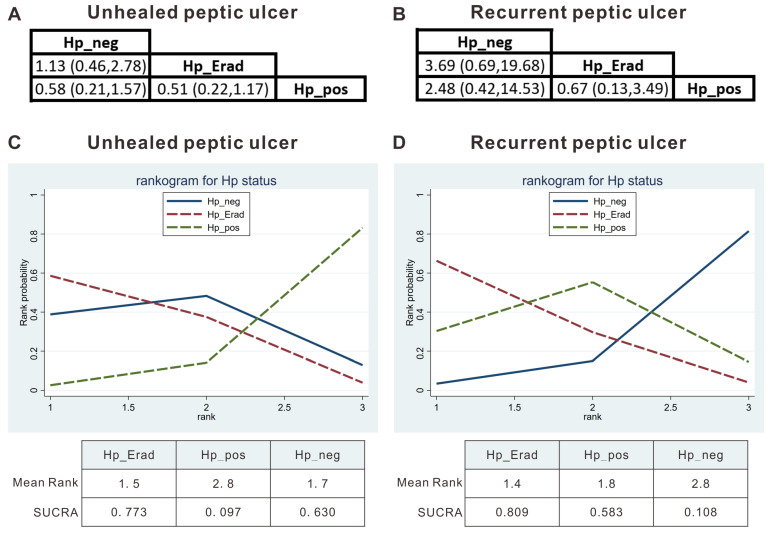
Summary of network meta-analysis results of pair-compare (**A**,**B**) and rankograms with surface under the cumulative ranking curve (SUCRA) (**C**,**D**).

No significant difference was detected between the effects of the Hp_pos group vs. Hp_neg group vs. Hp_Erad group by direct comparisons (Figure 2C). As mentioned, inconsistency tests were not feasible, and network meta-analysis estimations could not be performed. Similarly, the heterogeneity of the network meta-analysis comparisons could not be calculated (Figure 3A). The network meta-analysis showed that comparisons between each group did not yield significant results (Figure 4A). The Rankogram showed that the Hp_Erad group had the highest SUCRA value (0.773, mean rank: 1.5), followed by the Hp_neg group (SUCRA 0.63, mean rank 1.7), and Hp_pos group (SUCRA 0.097, mean rank 2.8; Figure 4C).

#### 3.6.2. Recurrent Peptic Ulcer

No significant difference was detected between the effects of the Hp_pos group vs. Hp_neg group vs. Hp_Erad group by direct comparisons (Figure 2D). As mentioned, inconsistency tests were not feasible, and network meta-analysis estimations could not be performed. Similarly, the heterogeneity of the network meta-analysis comparisons could not be calculated (Figure 3B). The network meta-analysis showed that comparisons between each group did not yield significant results (Figure 4B). The rankogram shows that the Hp_Erad group had the highest SUCRA value (0.809, mean rank: 1.4), followed by the Hp_pos group (SUCRA 0.583, mean rank 1.8), and Hp_neg group (SUCRA 0.108, mean rank 2.8; Figure 4D).

### 3.7. Other Results

Very few or no studies reported rebleeding of peptic ulcer, mortality, and safety of treatment, and these results were not available for further analysis in this network meta-analysis.

### 3.8. Comparison-Adjusted Funnel Plot

Risks of reporting and small-study effect biases affecting network meta-analysis of unhealed peptic ulcer and recurrent peptic ulcer were analyzed by visual assessment of the funnel plots (Supplementary Figure S2). The bias regarding unhealed peptic ulcers was not serious, but there could be some bias in recurrent peptic ulcers.

## 4. Discussion

Since the discovery of the association between *H. pylori* and peptic ulcer, the morbidity and mortality of peptic ulcers have decreased globally [26]. However, when peptic ulcer is complicated with liver cirrhosis, these patients have an increased hospital burden and higher in-hospital mortality compared with those not complicated with liver cirrhosis [27]. In fact, for cirrhotic patients, acute peptic ulcer bleeding had similar survival compared with acute variceal bleeding using similar therapies [28], without eradication of *H. pylori*. It is important to understand whether *H. pylori* is the predominant etiology of peptic ulcers in patients with liver cirrhosis to manage them optimally. This study did not identify a significant advantage of *H. pylori* eradication over persistent *H. pylori* infection or *H. pylori* infection in terms of improved healing or recurrence of peptic ulcers in cirrhotic patients. To our knowledge, this is the first network meta-analysis to assess the effects of *H. pylori* infection on the treatment outcomes of peptic ulcers in patients with liver cirrhosis.

The association of *H. pylori* with peptic ulcers in cirrhotic patients is still controversial. No specific recommendation regarding *H. pylori* eradication has been provided for those complicated with liver cirrhosis in recent consensus and guidelines [10,13,14,29]. The need for caution with the use of clarithromycin, tetracycline, metronidazole, and rifabutin for *H. pylori* eradication in patients with liver dysfunction has been addressed in only a single recent guideline [30]. Whether or not to treat *H. pylori* in cirrhotic patients is also a question, considering the increasing antibiotic resistance of *H. pylori* [31] and increasing trend in the recurrence rate of *H. pylori* infection [32]. In cirrhotic patients, who often require repeated antibiotic exposure for complications such as acute esophageal and gastric variceal bleeding, and spontaneous bacterial peritonitis, the risk of harboring resistant *H. pylori* strains may be particularly high [33], potentially reducing the efficacy of standard eradication regimens. Moreover, the safety of *H. pylori* eradication regimens is not completely understood in patients with abnormal liver function [16]. Additionally, in liver cirrhosis, recurrent peptic ulcer bleeding seemed to be related to cirrhosis per se but not *H. pylori* infection [34]. Of the 4 studies included in this meta-analysis, 3 demonstrated that *H. pylori* eradication did not reduce the recurrence rate of peptic ulcers in patients with cirrhosis [21,22,24]. Though some recent studies demonstrated that *H. pylori* infection contributed to peptic ulcer in liver cirrhosis, they only assessed symptomatic changes before and after eradication, rather than endoscopic confirmation of ulcer healing, which may have led to an overestimation of their conclusions [35]. In this study, we demonstrated that the endoscopic healing or recurrence of peptic ulcers in cirrhotic patients could not be significantly improved by the eradication of *H. pylori*. These results suggest that peptic ulcers in liver cirrhosis are not primarily related to *H. pylori* infection. Moreover, treatment of *H. pylori* should be determined carefully in cirrhotic patients because of impaired drug metabolism, vigilant for potential adverse effects [36].

The mechanism of peptic ulcer is complex in liver cirrhosis, which could be independent of *H. pylori* infection [37]. The use of nonsteroidal anti-inflammatory drugs (NSAIDs) could be one of the ulcerogenic factors in cirrhotic patients [38], but in our meta-analysis, all the enrolled studies excluded those taking NSAIDs. Other factors promoting peptic ulcer in liver cirrhosis include impaired mucosal defense, vascular dysfunction, thrombocytopenia, other bacterial infections, and renal dysfunction [28]. More importantly, clinically significant portal hypertension could induce mucosal ulceration and hemorrhage [28]. Portal hypertension could impair gastric mucosa by creating submucosal arteriovenous communication, inducing hyperemic response, and reducing the perfusion of gastric mucosa, and the levels of cytoprotective prostaglandin E2 as well as gastric mucin in gastric mucosa [39]. Propranolol which reduces portal pressure was linked to reduced recurrence of peptic ulcer bleeding in cirrhotic patients, which has not been observed with *H. pylori* eradication [28,34]. As some researchers also suggested that carvedilol, another drug which could reduce portal pressure, had a gastroprotective effect on aspirin-induced gastric damage and improved gastrointestinal symptoms in patients with ischemic heart disease on aspirin therapy [40,41], maybe future studies could compare the effects of portal pressure-lowering medications with eradication of *H. pylori* on the treatment outcomes of peptic ulcer in patients with cirrhosis.

Regarding clinical implications, according to the current results of the network meta-analysis, eradication of *H. pylori* seems to be unnecessary to improve healing or prevent recurrent peptic ulcer in patients with liver cirrhosis. However, given the physiological complexities of cirrhosis and the lack of specific recommendations in current guidelines for this population, our findings underscore the urgent need for further well-designed RCTs to confirm these findings.

This study has several limitations. First, only 4 prospective studies with relatively few patients were available for analysis. Second, the quality of the included studies was relatively low, primarily due to major concerns regarding imprecision. Furthermore, only healing and recurrence of peptic ulcer were reported most frequently, which could be analyzed in this study, and it is unknown whether other outcomes, such as ulcer bleeding, could be affected by *H. pylori* in cirrhotic patients. Moreover, because of the nature of the included studies, inconsistency did not exist, and thus, complex analysis could not be performed. These limitations are mostly due to the rigorous inclusion and exclusion criteria. However, our approach ensures a high degree of clinical homogeneity among the enrolled patients, enhances the internal validity of the findings, and reduces the risk of selection bias, thereby strengthening the reliability of the conclusions drawn from the synthesized evidence. These limitations are inherent to the current research landscape rather than flaws in study design. Despite non-significant results and these limitations, this study represents the first attempt to synthesize direct and indirect evidence on the effects of *H*. *pylori* on the treatment outcomes of peptic ulcer in cirrhotic patients, providing a comprehensive overview of current evidence and identifying critical gaps for future research. Thus, subsequent multicenter, large-scale RCTs assessing outcomes of peptic ulcers in patients with liver cirrhosis are needed.

## 5. Conclusions

Based on the available low-quality evidence, this network meta-analysis did not detect a statistically significant benefit of *H. pylori* eradication for ulcer healing or recurrence prevention in cirrhotic patients. These findings should be interpreted as highlighting an evidence gap rather than providing definitive evidence. Given the physiological complexities of cirrhosis and the lack of specific recommendations in current guidelines for this population, our findings underscore the urgent need for further well-designed RCTs to confirm these findings. Until such high-quality evidence is available, clinical decisions regarding *H. pylori* eradication in cirrhotic patients should be made on a case-by-case basis, weighing the potential benefits against the risks.

## Figures and Tables

**Table 1:** Characteristics of the included studies.

Author (Year)	Study Design	Population	Exclusion Criteria	Initial Tests of Hp	Testing for Eradication Success	Hp Eradication Therapy	Groups
Tzathas C. (2008) [24]	Prospective cohort study	Cirrhosis with peptic ulcer (duodenal and gastric)	(1) previous gastric surgery, (2) aspirin or nonsteroidal anti-inflammatory drug (NSAID) use within 2 months before study entry, (3) antisecretory drug (H2 receptor antagonist or proton pump inhibitor) use within 2 months before study entry, (4) history of *H. pylori* eradication therapy, (5) active variceal bleeding or stigmata of recent variceal bleeding at endoscopy, (6) hepatic encephalopathy, (7) hepatocellular carcinoma, (8) other debilitating diseases, that is, malignancy, heart or kidney failure, chronic obstructive pulmonary disease, and stroke	*H. pylori*-positive: either a positive urease test or a typical microscopic appearance.	*H. pylori*-eradicated: both rapid urease test and histologic examination results were negative.	Omeprazole 20 mg, amoxicillin 1 g, and clarythromycin 500 mg, all twice daily for 7 days. If the previous attempt failed, then a 2-week regimen with omeprazole 20 mg twice daily, bismuth subcitrate 125 mg 4 times daily, metronidazole 500 mg thrice daily, and tetracycline hydrochloride 500 mg 4 times daily.	Hp_pos Hp_Erad Hp_neg
Mitrică D. (2011) [22]	Prospective cohort study	Cirrhosis with peptic ulcer (duodenal and gastric)	Patients with a history of malignancy, gastric surgery, *H. pylori* eradication, presence of significant comorbidities, active variceal bleeding, hepatic encephalopathy and those who had taken PPIs, antibiotics, bismuth compounds and/or non-steroidal anti-inflammatory drugs four weeks before endoscopy	*H. pylori*-positive: both a positive urease test and a typical microscopic appearance.	*H. pylori*-eradicated: both rapid urease test and histologic examination results were negative.	Omeprazole (standard dose, twice a day) combined with amoxicillin (1.0 g twice a day) and clarithromycin (500 mg twice a day) for 1 week. No retreatment of *Helicobacter pylori* if the previous attempt failed.	Hp_pos Hp_Erad Hp_neg
Lo G. H. (2005) [21]	Prospective cohort study	Cirrhosis with duodenal ulcers	(1) malignancy, uremia, cerebrovascular accident, heart failure, chronic obstructive pulmonary disease, or other debilitating illness; (2) Child-Pugh’s scores O10; (3) taking NSAIDs or aspirin within 1 month before the initial endoscopy; (4) undergone antisecretory therapy, such as H2-receptor blocker or proton pump inhibitor within 3 months before the study; (5) had received eradication therapy of *H. pylori* before this study; (6) had previous gastric resection; (7) had bleeding from gastroesophageal varices or a peptic ulcer within 2 months before this study; and (8) unwilling to receive follow-up endoscopy.	*H. pylori*-positive: either a positive urease test or a typical microscopic appearance.	*H. pylori*-eradicated: both rapid urease test and histologic examination results were negative.	20 mg pantoprazole or 30 mg lansoprazole combined with 1000 mg amoxicillin and 500 mg clarithromycin twice a day for 1 week. No retreatment of *Helicobacter pylori* if the previous attempt failed.	Hp_pos Hp_Erad Hp_neg
Mo M. (2003) [23]	Prospective cohort study	Cirrhosis with peptic ulcer (duodenal and gastric)	Patients on bismuth, antibiotics or proton pump inhibitor within recent 1 month. Patients on long-term NSAIDs. Patients with pregnancy, lactation period, gastrinoma, severe encephalopathy, lung and heart diseases, or evidence of esophagogastric variceal bleeding	*H. pylori*-positive: both a positive urease test and a typical microscopic appearance.	*H. pylori*-eradicated: both rapid urease test and histologic examination results were negative.	20 mg omeprazole combined with 240 mg bismuth, 500 mg Tinidazole and 250 mg clarithromycin twice a day for 2 weeks. No retreatment of *Helicobacter pylori* if the previous attempt failed.	Hp_pos Hp_Erad

Hp, *Helicobacter pylori*. Hp_pos, *H. pylori* tested positive throughout the study (including those who failed to eradicate *H. pylori* and who denied eradication therapy). Hp_neg, *H. pylori* tested negative at baseline. Hp_Erad, *H. pylori* tested positive at baseline but successfully eradicated afterwards.

**Table 2:** Results of the included studies.

Author (Year)	Groups	Unhealed Peptic Ulcer		Recurrent Peptic Ulcer	
		e	n	e	n
Tzathas C. (2008) [24]	Hp_pos	0	1	0	1
	Hp_Erad	1	18	8	17
	Hp_neg	1	11	9	10
Mitrică D. (2011) [22]	Hp_pos	3	8	2	8
	Hp_Erad	4	14	2	13
	Hp_neg	7	17	10	15
Lo G. H. (2005) [21]	Hp_pos	3	18	8	18
	Hp_Erad	5	36	21	36
	Hp_neg	4	50	24	50
Mo M. (2003) [23]	Hp_pos	7	13	13	13
	Hp_Erad	19	63	39	63

Hp_pos, *H. pylori* tested positive throughout the study (including those who failed to eradicate *H. pylori* and who denied eradication therapy). Hp_neg, *H. pylori* tested negative at baseline. Hp_Erad, *H. pylori* tested positive at baseline but successfully eradicated afterwards.

## Data Availability

The data that support the findings of this study are available via https://data.mendeley.com/datasets/cwzsrxg8t4/1 (doi: 10.17632/cwzsrxg8t4.1), accessed on 18 February 2025.

## Supplementary Materials

The following supporting information can be downloaded at: https://www.mdpi.com/article/10.3390/jcm15062283/s1, Table S1: Newcastle–Ottawa Scale (NOS) for included studies. Table S2: Certainty of evidence evaluated with Confidence in Network Meta-Analysis (CINeMA) for unhealed peptic ulcer. Table S3: Certainty of evidence evaluated with CINeMA for recurrent peptic ulcer. Figure S1: Contribution plots. Figure S2: Funnel plots. Supplemental Material S1: PRISMA NMA checklist. Supplemental Material S2: Search strategy.
