# Supplementary material for: The Effects of Helicobacter pylori on the Treatment Outcomes of Peptic Ulcer in Patients with Liver Cirrhosis: A Systematic Review and Network Meta-Analysis
Source: J Clin Med. 2026 Mar 17;15(6):2283. doi: 10.3390/jcm15062283 (PMC13027053; doi:10.3390/jcm15062283)
Supplement: Supplementary file 1 [file jcm-15-02283-s001.zip › Supplemental Material S2 search strategy.pdf]

Supplemental Material S2

Search strategies

The search strategies are as follows.

For PubMed: (1) ((Helicobacter pylori eradication) AND (ulcer)) AND (cirrhosis); (2) (Helicobacter pylori) AND (hepatogenic ulcer).

For Ovid medicine: (1) (Helicobacter pylori eradication and ulcer and cirrhosis).af.; (2) (Helicobacter pylori and hepatogenic ulcer).af.

For Web of Science: (1) Helicobacter pylori eradication (Topic) and ulcer (Topic) and cirrhosis (Topic); (2) Helicobacter pylori (Topic) and hepatogenic ulcer (Topic).

For ClinicalTrials.gov: Condition or disease: Helicobacter pylori; Other terms: cirrhosis.
