# Supplementary material for: The Effects of Helicobacter pylori on the Treatment Outcomes of Peptic Ulcer in Patients with Liver Cirrhosis: A Systematic Review and Network Meta-Analysis
Source: J Clin Med. 2026 Mar 17;15(6):2283. doi: 10.3390/jcm15062283 (PMC13027053; doi:10.3390/jcm15062283)
Supplement: Supplementary file 1 [file jcm-15-02283-s001.zip › Table S1 NOS results.pdf]

Supplementary Table S1. Newcastle-Ottawa Scale (NOS) for included studies

**Author, year**	**Selection**	**Comparability**	**Outcome**	**NOS score**	**Quality (AHRQ standards)**
Tzathas C, 2018	4	1	3	8	Good
Mitrică D, 2011	4	1	3	8	Good
Lo GH, 2015	4	1	3	8	Good
Mo M, 2003	4	1	3	8	Good

AHRQ standards, USA Agency for Healthcare Research and Quality standards.
