# Supplementary material for: The Effects of Helicobacter pylori on the Treatment Outcomes of Peptic Ulcer in Patients with Liver Cirrhosis: A Systematic Review and Network Meta-Analysis
Source: J Clin Med. 2026 Mar 17;15(6):2283. doi: 10.3390/jcm15062283 (PMC13027053; doi:10.3390/jcm15062283)
Supplement: Supplementary file 1 [file jcm-15-02283-s001.zip › Table S3 CINeMA for recurrent peptic ulcer.pdf]

Supplementary Table S3. Certainty of evidence evaluated with CINeMA for recurrent peptic ulcer

Comparison	No. of studies	Within-study bias	Reporting bias	Indirectness	Imprecision	Heterogeneity	Incoherence	Confidence rating
Hp_Erad vs. Hp_neg	3	No concerns	Low risk	No concerns	Major concerns	No concerns	No concerns	Low
Hp_Erad vs. Hp_pos	4	No concerns	Low risk	No concerns	Major concerns	No concerns	No concerns	Low
Hp_neg vs. Hp_pos	3	No concerns	Low risk	No concerns	Major concerns	No concerns	No concerns	Low
