# Supplementary figures and images for: The Effects of Helicobacter pylori on the Treatment Outcomes of Peptic Ulcer in Patients with Liver Cirrhosis: A Systematic Review and Network Meta-Analysis
Source: J Clin Med. 2026 Mar 17;15(6):2283. doi: 10.3390/jcm15062283 (PMC13027053; doi:10.3390/jcm15062283)

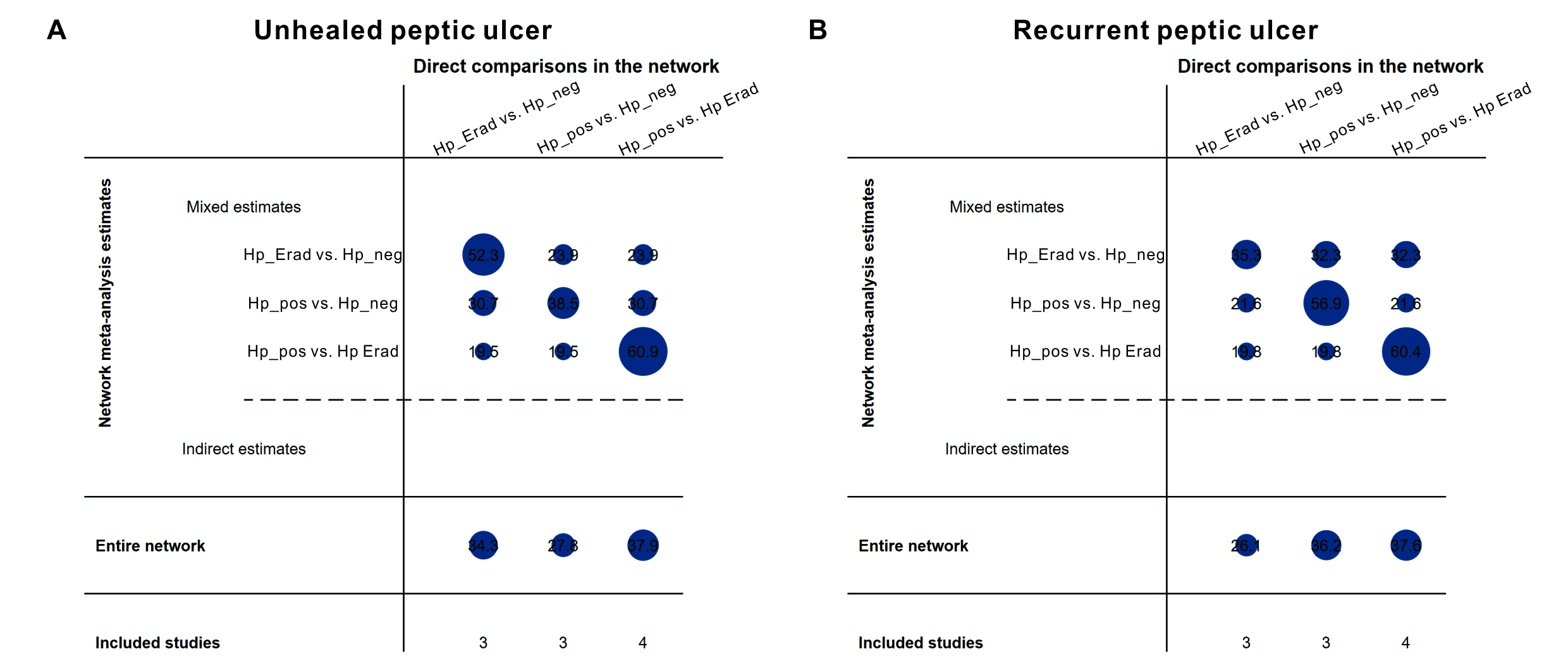

Supplement: Supplementary file 1 [file jcm-15-02283-s001.zip › Figure S1 Contribution plots.tif]

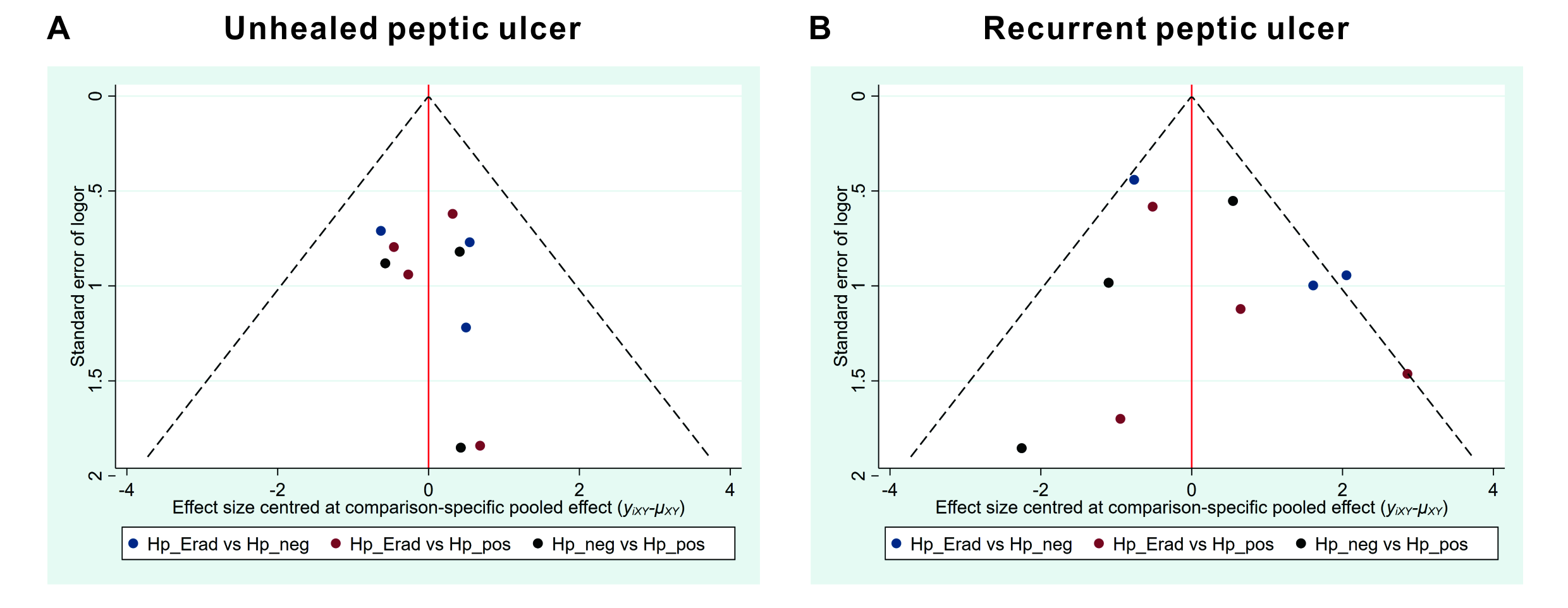

Supplement: Supplementary file 1 [file jcm-15-02283-s001.zip › Figure S2 funnel plot.tif]
